# Datasets of harmonized risk assessment of grapevine downy mildew and phenological observations in eight Italian regions (2012–2017)

**DOI:** 10.1016/j.dib.2022.108409

**Published:** 2022-06-23

**Authors:** Simone Bregaglio, Francesco Savian, Elisabetta Raparelli, Danilo Morelli, Rosanna Epifani, Gianni Fila, Luisa M. Manici

**Affiliations:** CREA - Council for Agricultural Research and Economics, Research Centre for Agriculture and Environment, I-40128 Bologna, Rome I-00184, Italy

**Keywords:** Plant diseases, BBCH codes, Expert-based assessment, Integrated pest management, Likert-scale

## Abstract

Phytosanitary bulletins released at weekly interval by eight Italian regional plant protection services in the growing seasons 2012–2017 were used to derive an harmonized dataset of grapevine downy mildew infection risk and phenological observations. The downy mildew infection risk (*n* = 8816) was classified using a 5-point Likert response item ranging from ‘very low’ (1) to ‘very high’ (5) by six independent evaluators with domain expertise in agronomy, phytopathology and agrometeorology. Common criteria have been used in the risk assessment, considering (i) the presence of disease symptoms in field surveys, (ii) the host phenological susceptibility, (iii) the weather forecasts in the next week from the bulletin release date, (iv) the advice to apply a fungicide treatment and (v) the outputs of epidemiological models. The phenological observations are provided as BBCH codes (*n* = 1689), which have been either transcribed from the phytosanitary bulletins or derived from the narrative description of the visual observation. Phenological data refer to the main early and late grapevine varieties in the eight regions (NUTS-2 administrative unit). Each record is associated with the NUTS-2 and NUTS-3 (31 provinces) administrative unit of reference, to the growing season (2012–2017), and refers to the individual risk assessment by the six evaluators. The dataset is hosted by the Centre for Agriculture and Environment of the Italian Council for Agricultural Research and Economics. These data could be helpful to researchers who develop either grapevine phenological models or process-based epidemiological predictive algorithms in order to refine their calibration and evaluation, as well as being a valuable resource for stakeholders in charge of evaluating the effective implementation of Integrated Pest Management in the decision-making process of public plant protection services in Italy. The dataset is freely available here.

## Specifications Table

**Table:** 

Subject	Agricultural Sciences: Agronomy and Crop Science
Specific subject area	Phytopathology, crop phenology and plant protection in the frame of Integrated Pest Management.
Type of data	Table
How the data were acquired	The downy mildew infection risk data were derived by six independent evaluators by consulting the phytosanitary bulletins delivered by eight Italian regional plant protection services in the period 2012–2017. Guidelines were defined to homogenize the classification of disease infection risk according to common criteria, and the infection risk was classified using a 5-point Likert response item, ranging from ‘very low’ (1) to ‘very high’ (5) risk. The grapevine phenological phases were reported as BBCH codes, which were either transcribed from the bulletins or inferred from the description of visual observations.
Data format	Raw
Description of data collection	Expert-based assessment of phytosanitary bulletins from six independent evaluators. The disease risk assessment data are categorized by NUTS-2 (region) and NUTS-3 (province) administrative unit, year (2012–2017) and evaluator (1–6). The phenological observations refer to the main early and late varieties in the grapevine cultivation areas.
Data source location	Institution: Research Centre for Agriculture and Environment, the Council for Agricultural Research and Economics City: Bologna Country: Italy
Data accessibility	Repository name: Mendeley Data Data identification number: 10.17632/3jsh4y2bw4.1 Direct URL to data: https://data.mendeley.com/datasets/3jsh4y2bw4/1
Related research article	S. Bregaglio, F. Savian, E., Raparelli, D., Morelli, R., Epifani, F., Pietrangeli, C., Nigro, R., Bugiani, S., Pini, P., Culatti, D., Tognetti, F., Spanna, M., Gerardi, I., Delillo, S., Bajocco, D., Fanchini, G., Fila, F., Ginaldi, L. M., Manici, A public decision support system for the assessment of plant disease infection risk shared by Italian regions. J. Environ. Manage. 2022, 26; 317–115,365, https://doi.org/10.1016/j.jenvman.2022.115365

## Value of the Data

• The release of a comprehensive dataset of grapevine downy mildew infection risk extends available knowledge on the implementation of Integrated Pest Management (IPM) principles in the operational decision-making process of public plant protection services in eight Italian regions (31 provinces), considering 2012–2017 growing seasons.
• The complementation of the dataset with grapevine phenological observations (1689 observations at NUTS-3 level) coded in the BBCH scale provides a solid basis to extend the calibration of phenological models as part of crop simulators for yield prediction, or to refine the predictive workflow of decision support systems for plant disease management.
• Researchers in the private or public sector working on the development and application of epidemiological models for plant disease risk forecasting can benefit from the availability of the robust, expert-based weekly assessment of grapevine downy mildew infections categorized in classes of risk ranging from ‘very low’ to ‘very high’ (5-point Likert response items).
• Researchers studying the variability of grapevine phenological development as a function of Environment × Management interactions can use this dataset to analyze differences in the timing of occurrence of phenological phases in the main grapevine varieties in the reference area over six years.
• Stakeholders in the public sector can use this dataset to assess the effective implementation of IPM principles in the decision-making process of plant protection services, concerning farmers support to optimize the application of fungicide treatments to counteract grapevine downy mildew, a major threat for grapevine production.
• The dataset can serve as the basis for an extended calibration and evaluation of the performances of process-based or empirical simulation models to reproduce either grapevine phenological development or downy mildew infection risk. Further insights could entail the estimation of the number of fungicide treatments advised by the plant protection services as a function of the assessment of grapevine downy mildew risk.

## 1 Data Description

The present dataset is organized in a table comprising 9007 records referred to an expert-based assessment of the risk of grapevine downy mildew infection released by Italian public plant protection services, and to grapevine phenological observations referred to eight regions (NUTS-2 administrative unit), 31 provinces (NUTS-3 administrative unit) and six years (2012–2017) (Table 1). The downy mildew infection risk (8816 records) is classified using an ordinal scale (5-point Likert response item) with the following levels: ‘very low’ (1), ‘low’ (2), ‘medium’ (3), ‘high’ (4), ‘very high’ (5). A consecutive numeric identifier ranging from 1 to 6 is assigned to the evaluators who performed the grapevine downy mildew risk assessment. Phenological observations (1689 records) are reported as BBCH codes [1]. When available from the phytosanitary bulletins used as the source of information, two phenological observations are reported, corresponding to the main early and late grapevine varieties in the respective cultivation areas.

**Table 1:** List of variables present in the dataset, their description and corresponding levels.

Variable	Description	Levels
NUTS_2	Italian Region	Abruzzo, Basilicata, Emilia-Romagna, Liguria, Lombardia, Marche, Sardegna, Veneto
NUTS_3	Italian Province	Ancona, Ascoli Piceno, Bergamo, Bologna, Brescia, Cagliari, Fermo, Ferrara, Forlì-Cesena, Genova, Imperia, L'Aquila, La Spezia, Macerata, Matera, Modena, Nuoro, Oristano, Parma, Pavia, Pesaro e Urbino, Piacenza, Potenza, Ravenna, Reggio nell'Emilia, Rimini, Sassari, Savona, Sondrio, Sud Sardegna, Venezia
Year	Grapevine season	2012, 2013, 2014, 2015, 2016, 2017
Date	Date	Date of release of the phytosanitary bulletin (m/dd/yyyy)
BBCH_late	BBCH code of late varieties	0, 1, 3, 5, 6, 7, 8, 9, 10, 11, 12, 13, 14, 15, 16, 17, 51, 53, 55, 57, 60, 61, 62, 63, 65, 67, 68, 69, 71, 73, 74, 75, 76, 77, 79, 80, 81, 83, 85, 86, 87, 89. NA = not available
BBCH_early	BBCH code of early varieties	
Evaluator	Evaluator identifier	1, 2, 3, 4, 5, 6. NA = not available
GDM_risk	Grapevine downy mildew risk	1, 2, 3, 4, 5. NA = not available

## 2 Experimental Design, Materials and Methods

Eight Italian regional plant protection services shared with the Research Centre for Agriculture and Environment of the Council for Agricultural Research and Economics (CREA-AA) the weekly phytosanitary bulletins which were released at provincial scale in the period 2012–2017. This dataset has been used to calibrate and evaluate the MISFITS-DSS [2].

Six independent evaluators including researchers and technicians working at CREA-AA with domain expertise in phytopathology, agronomy and agrometeorology read the bulletins with the objective of deriving the risk of grapevine downy mildew infection according to common criteria [2]. The disease infection was classified using a 5-point Likert response item [3] ranging from ‘very low’ to ‘very high’. Pre-defined common risk assessment criteria were followed by the six evaluators in the classification: (i) the presence of symptoms in field surveys; (ii) the host phenological susceptibility; (iii) the suitability of weather conditions for new infections in the following week; (iv) the advice of applying a fungicide treatment and (v) the model-based indication of downy mildew infection risk, when a simulation model was in use by the regional plant protection service.

Grapevine BBCH codes were either directly transcribed from the phytosanitary bulletins, or inferred from the description of the visual observation. When available, two observations were reported for the same bulletin, referred to the main early and late grapevine varieties in the cultivation areas. The BBCH codes present in the dataset are reported in Table 2.

**Table 2:** Grapevine growth stage, BBCH code and description corresponding to the phenological observations present in the dataset.

Grapevine growth stage	BBCH code	Description
0: Sprouting/Bud development	0	Dormancy: winter buds pointed to rounded, bright or dark brown according to cultivar, bud scales more or less closed according to cultivar
	1	Beginning of bud swelling: buds begin to expand inside the bud scales
	3	End of bud swelling: buds swollen, but not green
	5	“Wool stage”: brown wool clearly visible
	7	Beginning of bud burst: green shoot tips just visible
	9	Bud burst: green shoot tips clearly visible
1: Leaf development	11	First leaf unfolded and spread away from shoot
	12	Two leaves unfolded
	13	Three leaves unfolded
	14	Four leaves unfolded
	15	Fifth leaves unfolded (shoots 10 cm)
	16	Six leaves unfolded
	19	Nine or more leaves unfolded
5: Inflorescence emergence	53	Inflorescence clearly visible
	55	Inflorescence swelling, flowers closely pressed together
	57	Inflorescences fully developed, flowers separating
6: Flowering	60	First flowerhoods detached from the receptacle
	61	Beginning of flowering: 10% of flowerhoods fallen
	63	Early flowering: 30% of flowerhoods fallen
	65	Full flowering: 50% of flowerhoods fallen
	68	80% of flowerhoods fallen
	69	End of flowering
7: Development of fruits	71	Fruit set: fruits begin to swell, remains of flowers lost
	73	Berries groat-sized, bunches begin to hang
	75	Berries pea-sized, bunches hang
	77	Begin of berry touch
	79	Berry touch complete
8: Ripening of berries	81	Beginning of ripening
	83	Berries brighting in colour
	85	Softening of berries
	89	Berries ripe for harvest

**Fig. 1:**
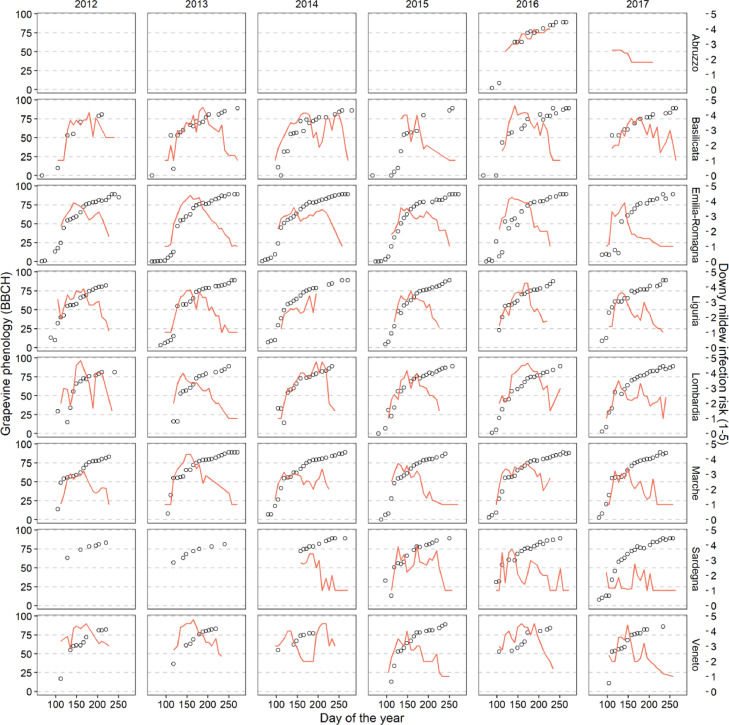
Average BBCH codes (black points, primary y-axis) and grapevine downy mildew infection risk (red line, secondary y-axis) in the eight NUTS-2 administrative units analyzed. Data are averaged by evaluators and NUTS-3 units within each NUTS-2 unit.

A graphical overview of the dataset is provided in Fig. 1, where BBCH codes and downy mildew infection risk are averaged by day of the year, evaluators and NUTS-3 administrative unit in the study area.

## Ethics Statement

These data are neither involved with human subjects, animal experiments nor obtained from social media platforms.

## CRediT authorship contribution statement

**Simone Bregaglio:** Conceptualization, Methodology, Investigation, Writing – original draft. **Francesco Savian:** Investigation. **Elisabetta Raparelli:** Investigation. **Danilo Morelli:** Investigation. **Rosanna Epifani:** Investigation. **Gianni Fila:** Investigation. **Luisa M. Manici:** Methodology, Investigation.

## Declaration of Competing Interest

The authors declare that they have no known competing financial interests or personal relationships that could have appeared to influence the work reported in this paper.

## Data Availability

Datasets of harmonized risk assessment of grapevine downy mildew and phenological observations in eight Italian regions (2012–2017) (Original data) (Mendeley Data).
